# Exploring Climate-Driven Mismatches Between Pollinator-Dependent Crops and Honeybees in Asia

**DOI:** 10.3390/biology14030234

**Published:** 2025-02-25

**Authors:** Ehsan Rahimi, Chuleui Jung

**Affiliations:** 1 Agricultural Science and Technology Institute, Andong National University, Andong 36729, Republic of Korea; ehsanrahimi666@gmail.com; 2 Department of Plant Medical, Andong National University, Andong 36729, Republic of Korea

**Keywords:** pollinators, pollination, species distribution modeling, species interactions, plant-pollinator mismatch

## Abstract

In Asia, *Apis cerana* (native) and *Apis mellifera* (introduced) are the primary managed honeybee species, vital for pollination and honey production. However, climate change and other threats are driving pollinator declines, while research on their ecology in Asia remains limited. Bridging these knowledge gaps is crucial for developing conservation strategies to sustain pollination services and agricultural systems in the region. In this study, we evaluated the potential impacts of climate change on the spatial interactions between two honeybee species (*A. cerana* and *A. mellifera*) and 20 pollinator-dependent crops across 23 countries in Asia. We used species distribution models (SDMs) to generate habitat suitability maps for both honeybees and crops under current and future climate scenarios (SSP585 for 2070). Schoener’s D statistic was employed to quantify the spatial overlap, and a novel spatial approach was applied to create mismatch maps that identified areas of increased or decreased interactions. We found that, on average, *A. cerana* demonstrated higher overlap with 12 crops compared to 8 for *A. mellifera* in future projections. Key crops like sesame, eggplant, and mango retained strong overlaps with both pollinators, while mismatches were more pronounced for *A. cerana*, particularly with watermelon, strawberry, and buckwheat. In contrast, *A. mellifera* showed greater stability and resilience in spatial overlaps with crops such as soybean and sunflower. Overall, *A. cerana* is expected to face more significant spatial mismatches, highlighting its vulnerability to climate-induced changes and the critical need for targeted conservation efforts. This study highlights the urgent need for region-specific conservation strategies by identifying areas at risk of losing critical pollination services. Our results provide a crucial framework for informed management and policy interventions to safeguard pollination-dependent agricultural systems and biodiversity in Asia amidst accelerating environmental challenges.

## Simple Summary

This study evaluated the impacts of climate change on the spatial interactions between two honeybee species and 20 pollinator-dependent crops across 23 countries in Asia. Using species distribution models (SDMs), habitat suitability maps were generated under current and future climate scenarios (SSP585 for 2070). Schoener’s D statistic quantified spatial overlaps, and mismatch maps identified areas of increased or decreased interactions. The results showed that *A. cerana* had higher overlap with 12 crops, while *A. mellifera* overlapped more with 8 crops under future projections. *A. mellifera* showed greater stability and resilience, with stable overlaps for crops such as soybean and sunflower. The findings highlight the vulnerability of *A. cerana* to climate-induced changes and emphasize the need for targeted conservation efforts. By identifying areas at risk of losing pollination services, this study provides a framework for region-specific management strategies to support pollination-dependent agriculture and biodiversity in Asia amidst environmental challenges.

## 1. Introduction

Pollinators, particularly insects, play a critical role in global food systems by facilitating the reproduction of 88% of flowering plants and contributing to the production of approximately 75% of the world’s food crops [1,2]. Their economic contribution to agriculture is valued from USD 195 to USD 387 billion annually [3]. Beyond economic metrics, pollinators are essential for human nutrition, supplying up to 40% of the dietary intake of vital nutrients such as vitamins A and C, antioxidants, and other micronutrients [4]. However, the agricultural systems are becoming increasingly dependent on pollinators, with evidence of significant shortages already emerging. For instance, a study in China revealed a rapid increase in agricultural reliance on pollinators between 1961 and 2018, with the demand for honeybee colonies exceeding the available stocks by threefold [5].

The decline of pollinators is a growing global concern, with habitat loss, pesticide exposure, diseases, and invasive species identified as major threats [6]. Among these, climate change poses a particularly insidious risk, disrupting plant–pollinator relationships and exacerbating the existing pressures [7]. Climate change can affect bees’ physiology, immune system, range, and access to floral resources, potentially leading to mismatches in resource availability [8,9,10,11]. Changes in precipitation patterns, for example, can alter the availability of floral resources, influencing pollinator movement and survival [12]. Temperature rise directly affects bee foraging behavior and physiological performance [13]. Rising temperatures are compelling many bee species to migrate towards higher latitudes and altitudes, leading to pollinator range shifts [14,15,16]. In this regard, some crops may experience reduced pollination services [17], especially if key pollinators move to areas where those crops are not grown or if the timing of flowering and pollinator activity becomes misaligned. Also, if pollinators such as *A. mellifera* shift their range or decline in abundance due to climate change, pollination deficits may arise, particularly for crops like apple (*M. domestica*) that rely on specific pollinator species [18].

One of the most critical consequences of climate change for plant–pollinator interactions is the emergence of temporal and spatial mismatches [19]. Temporal mismatches occur when the flowering periods of plants and the activity periods of their pollinators are no longer synchronized, reducing pollination efficiency and crop yields [20,21]. For example, numerous studies also indicate that the rising spring temperatures have led to earlier flowering in many plant species. For example, since the 1960s, spring-related events, such as vegetation growth and flowering, have consistently occurred earlier [22,23,24,25,26,27,28,29,30]. However, plants often struggle to shift their ranges at comparable rates, with migration rates as low as 20–40 km per century, creating significant spatial mismatches [31].

Spatial mismatches arise when changes in the climatic conditions drive shifts in the geographic ranges of pollinators and plants at different rates [32]. Species distribution models predict significant declines in bumblebee diversity in Europe by 2050, with only mountainous regions retaining substantial populations by 2100 [33]. These mismatches can lead to the breakdown of mutualistic networks, potentially forming new but less efficient plant–pollinator associations [34,35].

The implications of these disruptions extend beyond the ecological systems, directly affecting human livelihoods and food security. Pollinator-dependent crops are critical for the production of essential nutrients, with over 90% of global vitamin C, b-cryptoxanthin, and b-tocopherol sourced from these plants [4]. Without pollinators, global crop production could decline by 3–8%, with the greatest impacts observed in developing countries where diets are highly reliant on pollinator-supported foods [36]. In a study by Rahimi et al. [37], the impact of climate change on the climate suitability of 61 pollinator-dependent crops globally was assessed using species distribution models (SDMs). The findings showed notable regional differences in habitat suitability for crops. By 2070, the suitable areas are expected to decline for 16 crops in Africa, 31 in Asia, 34 in Australia, 29 in Europe, 29 in North America, and 31 in South America. On average, climate suitability is projected to decrease by 14.5% in Africa, 11.2% in Asia, 26.2% in Australia, 4.7% in Europe, and 14.4% in South America, while North America is expected to see a slight increase of 5.5%.

Pollinating insects represent a diverse array of taxa, including bees, butterflies, moths, hoverflies, and other dipteran species. Among these, bees play a particularly pivotal role, contributing to the pollination of plants allowing for approximately 35% of the world’s food production [38]. Bees, being ectothermic organisms, depend heavily on ambient temperature for their metabolic functions and activity levels. Their contributions to global agriculture are unparalleled, with honeybees and bumblebees visiting over 90% of food crops worldwide [39]. The western honeybee (*A. mellifera*), in particular, is recognized as the most frequent floral visitor globally, as highlighted by Hung et al. [40]. Their study, which analyzed 80 plant–pollinator networks and the pollination effectiveness for 34 plant species, found that *A. mellifera* accounted for an average of 13% of floral visits in natural habitats, ranging from 0% to 85%. Remarkably, 5% of the plant species depended exclusively on this bee species, which was the dominant visitor for 33% of the networks and 49% of the plant species, underscoring its ecological importance.

While studies in Europe and North America have assessed the spatial alignment of pollinator-dependent crops and bee species using SDMs, similar efforts in Asia are notably absent. For example, analyses in Europe and North America have mapped 394 and 697 bee species, respectively, alongside 41 pollinator-dependent crops [41]. These studies revealed significant regional differences, with Europe showing a higher overlap between bees and crops compared to North America. However, the absence of such studies in Asia represents a critical gap, leaving this region with limited data to guide pollination management and conservation strategies.

Asia is home to nine honeybee species, including *Apis dorsata*, *Apis laboriosa*, *Apis florea*, *Apis reniformis*, *A. cerana*, and the introduced *A. mellifera* [42,43]. Among these, only *A. cerana* and *A. mellifera* are managed in hives for honey production and pollination, while the other species are traditionally harvested through honey-hunting practices [43]. *A. cerana*, the native Asian honeybee, and *A. mellifera*, imported for commercial honey production, are the primary managed species in the region. Despite their importance, research and conservation efforts for honeybees and other pollinators in Asia face significant challenges [44,45]. Warrit et al. [46] emphasized the need for locally tailored solutions to address these challenges, particularly for less-studied species. However, the knowledge gaps in Asia, particularly concerning species distributions, ecological roles, and threats, remain far greater than in other regions. This lack of information hinders effective conservation and management strategies, threatening both pollinator populations and the ecosystems they support [46,47].

Asia’s diverse honeybee species and increasing dependence on pollinator-driven agriculture underscore its importance as a focal region for research and conservation [48]. Bridging the significant knowledge gaps and crafting regionally specific strategies are critical to ensuring the resilience of pollination services amidst mounting environmental challenges. This study seeks to address these gaps by evaluating the impacts of climate change on the distributions of two key honeybee species (*A. cerana* and *A. mellifera*) and 20 pollinator-dependent crops across Asia. Specifically, we aim to predict how climate change may influence the future distribution of both honeybees and crops individually, as well as their interactions. The study identifies regions where honeybee–crop interactions are likely to increase or decrease under changing climatic conditions, providing an essential framework for identifying areas at risk of losing these interactions. This knowledge will be instrumental in guiding proactive management strategies to mitigate risks and ensure the long-term sustainability of pollination services in Asia.

## 2. Materials and Methods

### 2.1. Honeybee Occurrence Data

**Figure 1:**
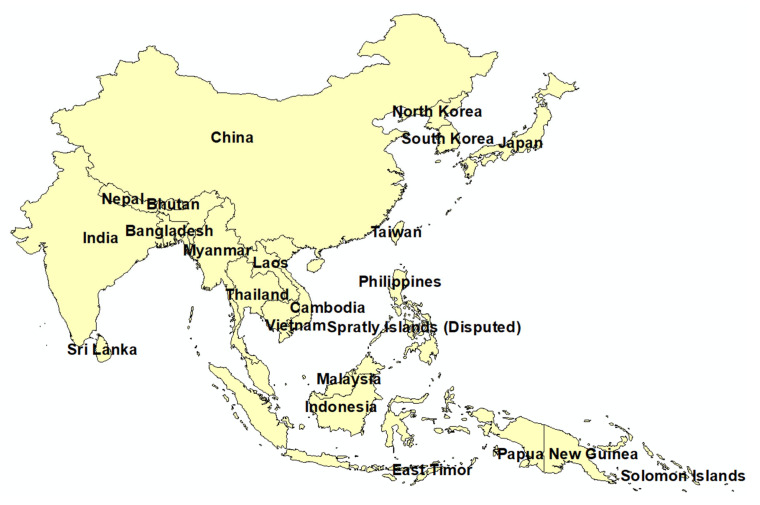
The location of the study area.

Asia hosts a remarkable diversity of honeybee species, with at least eight native species, most of which are concentrated in tropical regions [42,44,49]. For our study area (Figure 1), we focused on two species: the native *A. cerana* and the non-native *A. mellifera*. These species were chosen because of their wide distribution and representativeness of other *Apis* species, as well as their relevance to current research. Other species, such as *A. dorsata* and *A. laboriosa*, have already been modeled in prior studies examining the impacts of climate change [50]. Incorporating these two honeybee species also aligns with the recognition of the role of highly social species as flagship representatives, which can raise awareness about lesser-studied groups [46,51,52]. Presence data for *A. cerana* and *A. mellifera* were retrieved from the Global Biodiversity Information Facility (GBIF; www.gbif.org). To mitigate spatial biases in the occurrence data, we applied spatial filtering using the “Humboldt” R package, ensuring a minimum distance threshold of 35 km between occurrence points across continents [53]. Our study area encompassed 23 countries, representing a diverse and ecologically significant region of Asia and the Pacific. These countries included Bangladesh, Bhutan, Brunei, Cambodia, China, East Timor, India, Indonesia, Japan, Laos, Malaysia, Myanmar, Nepal, North Korea, Papua New Guinea, Philippines, Solomon Islands, South Korea, Spratly Islands (disputed), Sri Lanka, Taiwan, Thailand, and Vietnam (Figure 1).

### 2.2. Crop Occurrence Data

The reliance of crops on pollination varies significantly and is classified into four distinct categories based on the degree of dependence [2]. These categories are defined as follows: (1) essential, where a lack of pollination reduces crop production by over 90%; (2) high, where production declines range between 40% and 90%; (3) modest, with production decreases of 10% to 40%; (4) little, where reductions are between 0% and 10%. For this study, the focus was placed on the first three groups of crop dependency on pollination. In total, 20 pollinator-dependent crops were selected for analysis, including one crop in the essential category, seven in the high-dependence category, and twelve in the modest-dependence category (Table 1). Occurrence data for these crops were obtained from the Global Biodiversity Information Facility (GBIF; www.gbif.org). To ensure compatibility with the bee occurrence data, a spatial thinning procedure was implemented, applying a minimum distance threshold of 35 km between occurrence points for the crop data.

### 2.3. Environmental Variables

The bioclimatic layers used as predictor variables were sourced from the WorldClim database (www.worldclim.org). The WorldClim dataset provides 19 bioclimatic variables, including 11 temperature-related and 8 precipitation-related variables, with a spatial resolution of approximately 4 km^2^. Since these variables are often highly correlated, it is generally not recommended to include all of them in species distribution modeling. To address this issue, we utilized the usdm package [54] to eliminate highly correlated variables through a stepwise procedure based on the Variance Inflation Factor (VIF). After this process, six bioclimatic variables were selected for the analysis: Mean Diurnal Range (Bio 2), Temperature Seasonality (Bio 4), Max Temperature of Warmest Month (Bio 5), Mean Temperature of Wettest Quarter (Bio 8), Precipitation of Wettest Quarter (Bio 12), and Precipitation of Driest Quarter (Bio 17). These variables were chosen for their reduced multicollinearity and relevance to the modeling species distributions.

### 2.4. Model Fitting and Assessment

To model the distribution of the studied species, we used the “FLEXSDM” R package [55], which incorporates the MaxEnt model to create climate suitability maps based on presence and climate data. The MaxEnt model, widely recognized for its application in species distribution modeling and assessing climate change impacts, is particularly effective in insect studies [56]. For the MaxEnt modeling process, we generated 10,000 random pseudo-absence points to complement the presence points on each continent. The climate suitability values range from 0 to 1, where 1 indicates maximum suitability. To project the future distribution of the studied species in 2070, we utilized the SSP585 climate change scenario, a shared socio-economic pathway representing an extreme climate trajectory. This scenario predicts a tripling of CO_2_ emissions by 2075 and a mean temperature rise of 4.4 degrees Celsius by 2070 [57]. By examining the most severe scenario, we sought to identify the largest possible changes in crop and honeybee distributions, enabling policymakers and researchers to anticipate and plan for significant climate-related impacts.

To evaluate the model performance, we used three metrics: Inverse Mean Absolute Error (IMAE), Area Under the ROC Curve (AUC), and Boyce Statistic (BOYCE), implemented via the “FLEXSDM” R package v1.3.5 [55]. AUC values between 0.7 and 0.9 indicate acceptable performance, while values above 0.9 reflect excellent predictions. IMAE, calculated as 1 − Mean Absolute Error, increases with model accuracy. The Boyce Index, which ranges from −1 to 1, assesses the alignment between observed and predicted probabilities, with values near 1 indicating strong agreement, and values near 0 suggesting random performance. We applied 5-fold cross-validation [58] for robust model evaluation.

### 2.5. Spatial Overlap Measuring

We used Schoener’s D [59,60] to measure the geographic distribution overlap between honeybees and crops under current and future climate scenarios. Using the *calc.Niche.Overlap* function from the ENMeval R package [61], we calculated spatial overlap values for various crop and honeybee pairs across Asia. We utilized this metric to quantify the spatial overlap between honeybees and crops under current and future scenarios. Subsequently, we compared the changes in this metric across the two periods to evaluate how spatial matching evolves. The D statistic ranges from 0 to 1, where 0 represents no spatial overlap, and 1 indicates complete overlap (Equation (1))

D (px, py) = 1 − 1/2 ∑│px, i − py, i│(1)

where px, i (or py, i) denotes the probability assigned by the SDMs for the species x (or y) to cell i.

### 2.6. Honeybee–Crop Mismatch Mapping

Schoener’s D metric provides a quantitative measure of spatial overlap between honeybees and crops under different climate scenarios. However, as this metric outputs a single numerical value rather than a map, it does not allow for the spatial identification of areas where interactions between honeybees and crops may increase or decrease. Since one of the goals of this study was to determine the specific regions in Asia where these interactions might change, a spatial approach was necessary. To achieve this, we utilized SDMs or habitat suitability maps for each species of honeybee and crop under current and future climate scenarios, obtaining 44 maps (22 species × 2 scenarios). Each map’s suitability values ranged from 0 to 1, representing the likelihood of occurrence for a species in a given cell. To calculate the interaction probability between a crop and a pollinator, we multiplied the suitability maps for each pair. If a cell in both maps had a suitability value of 1, the resulting cell in the output map also received a value of 1, indicating full spatial overlap. Conversely, if either cell had a value of 0, the resulting cell was assigned a value of 0, indicating no overlap in that location.

This process was repeated for all pairs of crops and honeybees. Since there were two honeybee species and 20 crops, we created 40 interaction maps for the current scenario and 40 for the future scenario (details available at https://github.com/ehsanrahimi666/Crop-honeybees.git, accessed on 1 February 2025). By subtracting the future interaction map from the current interaction map for each pair, we generated a “spatial mismatch map”. Positive values in these maps indicate areas where the probability of interaections between the crop and the pollinator would increase under future climate conditions, while negative values represent areas likely to lose interactions. This approach allowed us to identify and visualize the spatial dynamics of honeybee–crop interactions across two time periods, providing valuable insights to locate areas of potential gain or loss in these critical ecological interactions. These spatial mismatch maps were created for all crop–honeybee pairs, offering a comprehensive view of how climate change might alter pollinator–crop relationships in Asia.

## 3. Results

### 3.1. Model Assessment

Table 2 presents the validation metrics for honeybees (*A. mellifera* and *A. cerana*) and pollinator-dependent crops using the MaxEnt algorithm. The metrics included AUC (Area Under the Curve), BOYCE (Boyce Index), and IMAE (Inverse Mean Absolute Error), which collectively evaluated the model performance. The AUC values ranged from 0.87 to 0.98, indicating strong predictive accuracy for most species. The BOYCE values were generally high, within a range from 0.87 to 0.99, reflecting strong agreement between observed and predicted suitability. The IMAE scores ranged from 0.73 to 0.96, with higher values indicating better model accuracy.

### 3.2. Overlap Between Honeybees and Crop Distribution

**Figure 2:**
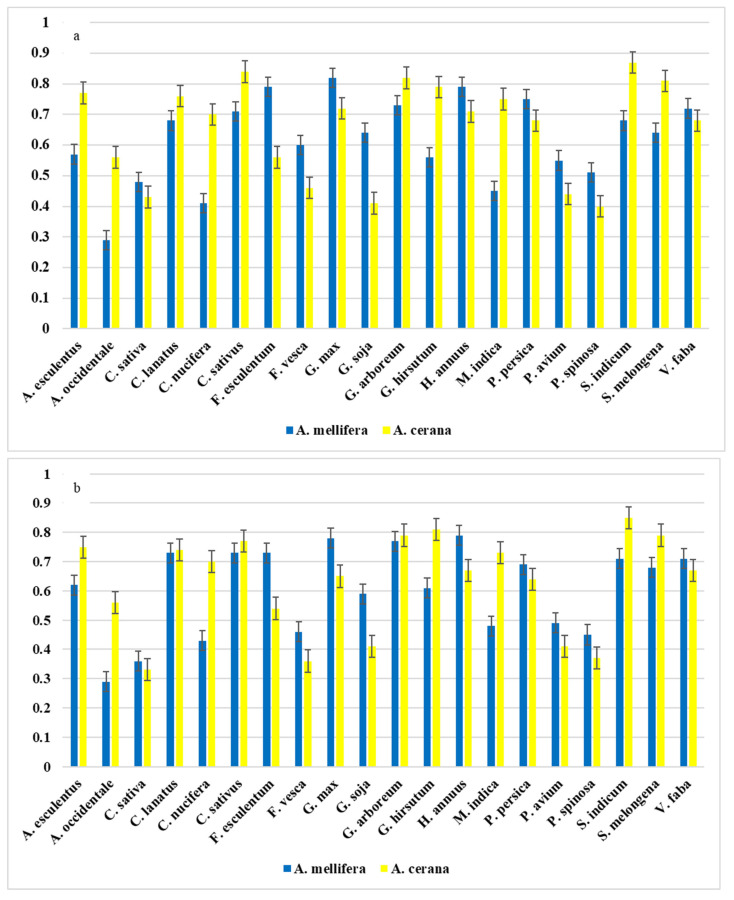
Spatial overlap between honeybees and crops in Asia based on Schoener’s D statistic in current (**a**) and future (**b**) scenarios.

Figure 2 presents an analysis of how much overlap exists between honeybees and pollinator-dependent crops in Asia under current and future climate conditions. The overlap was measured using Schoener’s D statistic, which ranges from 0 to 1, with values closer to 1 indicating a high degree of shared habitat for the studied species, meaning that both the bee species and the crop are likely to be found in the corresponding areas. Under the current climate, *A. cerana* showed a higher overlap with 11 crops, while *A. mellifera* had a higher overlap with 9 crops. For *A. cerana*, the highest overlap values were found with *S. indicum* (0.87), *S. melongena* (0.81), *C. sativus* (0.84), *C. lanatus* (0.76), and *M. indica* (0.75). This means that these crops and *A. cerana* are highly likely to be found in the same areas, making *A. cerana* an important pollinator for them. In comparison, *A. mellifera* showed the highest overlap with *G. max* (0.82), *F. esculentum* (0.79), *H. annuus* (0.79), *G. arboreum* (0.73), and *P. persica* (0.75). Some crops had similar overlap values for both bee species, for example, *P. avium* had an overlap of 0.55 with *A. cerana* and 0.44 with *A. mellifera*, while *P. spinosa* had an overlap of 0.51 with *A. cerana* and 0.40 with *A. mellifera*. This means that both bee species may be present in the same regions for these crops, but *A. cerana* is slightly more common.

Under the future climate scenario (SSP585), *A. cerana* was projected to have a higher overlap with 12 crops, while *A. mellifera* will have a higher overlap with 8 crops. The top five crops with the highest overlap for *A. cerana* remained similar to those identified under the current climate, with values of 0.85 for *S. indicum* 0.85, 0.79 for *S. melongena*, 0.79 for *G. arboreum*, 0.74 for *C. lanatus*, and 0.73 for *M. indica*. This suggests that despite the climate change, *A. cerana* will still share a significant range with these crops, although some minor shifts may occur. For *A. mellifera*, the top five overlap values were measured for *H. annuus* (0.79), *G. max* (0.78), *G. arboreum* (0.77), *C. sativus* (0.73), and *F. esculentum* (0.73), showing that this species will remain an important pollinator for these crops, with its distribution overlapping theirs quite consistently. Climate change is expected to cause shifts in pollination dynamics, with some crops experiencing more stable interactions, while others seeing reductions in pollinator overlap. For *A. mellifera*, the overlap with most crops will remain stable or decrease slightly under the future scenario. For example, the overlap with *C. lanatus* slightly increased from 0.68 to 0.73, while that with *A. esculentus* rose from 0.57 to 0.62. However, some crops such as *G. max* (0.78 to 0.77) and *G. arboreum* (0.77 to 0.73) experienced minor declines, which could indicate changes in pollination availability in certain regions. Interestingly, *A. mellifera* showed a stable or slightly increased overlap with crops found in tropical regions, such as *C. nucifera*, suggesting that it may be more adaptable to areas where climate conditions will remain relatively stable.

For *A. cerana*, however, some notable declines in overlap were observed under climate change. For instance, its overlap with *C. lanatus* dropped from 0.76 to 0.74, while that with *F. vesca* decreased more significantly from 0.46 to 0.36. This suggests that *A. cerana* could face challenges in maintaining interactions with certain crops in areas affected by climate stressors. However, not all interactions declined; some crops, such as *S. indicum*, continued to have high overlap values with *A. cerana*, which decreased only slightly from 0.87 to 0.85, indicating that *A. cerana* may still play a key pollination role for these crops despite climate change. Examining specific crop–pollinator interactions revealed that certain crops will experience increased overlap with one pollinator, while reporting decreased overlap with the other. For example, *C. nucifera*, *C. sativus*, and *H. annuus* showed slight increases or stability in their overlap with *A. mellifera*, indicating that they may be better suited to future climate conditions. On the other hand, *A. cerana* showed slight declines in the overlap with these same crops, particularly in areas expected to become less favorable due to changing climate conditions. Both bee species showed reduced overlap with *F. vesca*, *F. esculentum*, and *G. max*, suggesting that these crops may face pollination challenges under future climate scenarios. These results highlight the complex ways in which climate change may influence the interactions between pollinators and crops, with some species being more adaptable while others may experience greater vulnerability.

### 3.3. Spatial Mismatch

**Figure 3:**
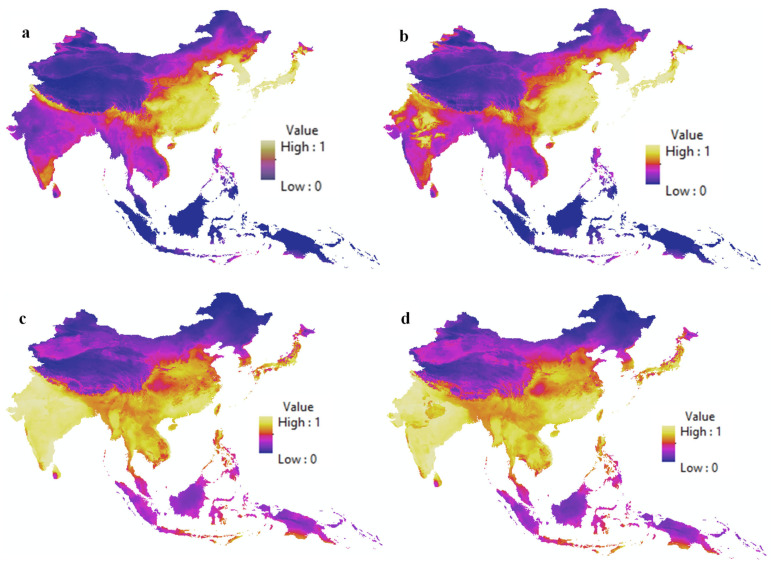
Habitat suitability maps illustrating the predicted distributions of *A. mellifera* under current (**a**) and future (**b**) climate scenarios and of *C. lanatus* under current (**c**) and future (**d**) scenarios.

**Figure 4:**
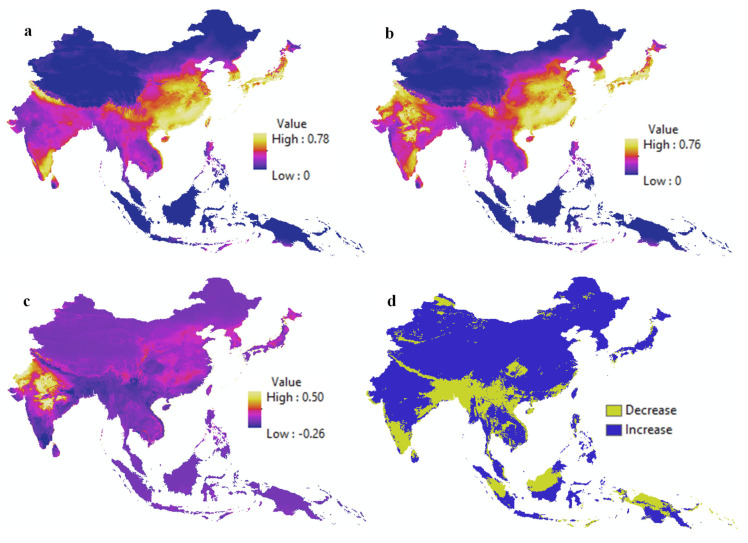
Interaction probability maps for *A. mellifera* and *C. lanatus* under current (**a**) and future (**b**) climate scenarios, their subtraction map showing changes in interaction probabilities (**c**), and the classified subtraction map highlighting areas of increased and decreased interactions (**d**).

Figure 3 presents habitat suitability maps for *A. mellifera* under current and future climate scenarios, as well as for *C. lanatus* under the same conditions. These maps show the areas where each species is likely to thrive, allowing for a visual comparison of their distribution between the present and 2070 under the SSP585 climate scenario, which represents the most extreme projections of climate change. A comparison of panels (a) and (b) shows that suitable habitats for *A. mellifera* are expected to expand in northern India by 2070, indicating a potential shift in the species’ range as a response to climate change. Figure 4 illustrates the interaction probability maps for *A. mellifera* and *C. lanatus* under both current and future climate conditions. Panels (a) and (b) show the overlap of the habitat suitability maps for *A. mellifera* and *C. lanatus*, representing areas where both species are likely to coexist and interact. A noticeable increase in interaction probability is observed in northern India under future climate conditions, which is indicated by the yellow-colored regions in the panel (b). In contrast, areas with a decline in interaction probability appear in blue.

Panel (c) displays a subtraction map, which was created by subtracting the future interaction probability map (panel b) from the current interaction probability map (panel a). This highlights areas across Asia where interactions between *A. mellifera* and *C. lanatus* are expected to either increase (positive values) or decrease (negative values) under climate change scenarios. To improve the interpretation, panel (d) presents a simplified classification of the subtraction map, dividing the regions into two categories: areas where the interactions are predicted to increase, and areas where they are projected to decline. This figure provides a case study of interaction dynamics between a single pollinator and a crop pair. The same methodology was applied to all crop–pollinator pairs, producing spatial mismatch maps that illustrate how interactions may shift due to climate change. These additional results are included in the Supplementary Materials for further reference. This spatial modeling approach offers valuable insights into how ecological interactions may change in response to future climate conditions, aiding in the understanding and management of pollination dynamics.

Table 3 provides a comprehensive analysis of the spatial mismatches between *M. indica* and two key honeybee species, *A. cerana* and *A. mellifera*, across 22 Asian countries under the future climate change scenario SSP585. The table records the number of raster cells where interaction probabilities between mango and these honeybee species are expected to increase or decrease. For *A. cerana*, the results showed a general trend of a higher increase in the number of cells with higher interactions compared to the number of cells with decreased interactions across most countries. Notably, China demonstrated the highest number of increased-interaction cells for *A. cerana*, with 30,778 increased-interaction cells compared to 3316 cells with decreased interactions. Similarly, India, a major mango-producing country, exhibited a significant number of increased-interaction cells (3438), despite having 6472 decreased-interaction cells. For *A. mellifera*, the patterns were more variable. While some countries showed substantial increases in interaction cells, others exhibited significant decreases, highlighting potential vulnerabilities for this species. China again showed a strong positive trend, with 30,682 increased-interaction cells, but also experienced 3412 decreased-interaction cells, indicating localized declines in interaction probabilities. India demonstrated notable increases in high-interaction cells (7198), the highest registered for *A. mellifera*, but also declines in interaction in 2712 cells, suggesting that this species may be more affected by regional climatic variations. Countries such as Myanmar and Bangladesh revealed higher numbers of decreased-interaction cells, with Myanmar showing 1368 decreased-interaction cells compared to 685 increased-interaction cells, and Bangladesh recording 424 decreased-interaction cells versus only 1 increased-interaction cell. These findings suggest potential risks to mango pollination services in these regions.

Certain countries, such as Indonesia and Thailand, exhibited strong positive trends for both honeybee species. Indonesia showed 3327 increased-interaction cells for *A. cerana* and 2997 for *A. mellifera*, while Thailand recorded 1239 increased-interaction cells for *A. cerana* and 920 for *A. mellifera*. Conversely, smaller countries such as Brunei, East Timor, and the Solomon Islands showed limited overall changes due to their smaller geographic extents, but localized shifts in pollination interactions were still evident. Overall, *A. cerana* demonstrated greater resilience in maintaining and expanding its spatial interactions with mango across Asia, likely due to its native status and broader adaptability to climatic variations. In contrast, *A. mellifera* appeared to be more susceptible to declines in its distribution in certain regions, particularly in countries like Myanmar and Bangladesh. These differences underline the importance of considering both native and non-native pollinators when planning for future mango cultivation and pollination strategies. Here, we selected mango as an example due to its significance in Asia, with similar analyses for other crops provided in the Supplementary Materials.

## 4. Discussion

In this study, we investigated the spatial interactions between honeybees (*A. mellifera* and *A. cerana*) and 20 pollinator-dependent crops across Asia under current and future climate scenarios (SSP585 for 2070). Using SDMs, we generated habitat suitability maps for both honeybees and crops, assessed their spatial overlaps, and quantified changes in these interactions under climate change. Our results showed that under the SSP585 climate change scenario, *A. cerana* is expected to experience a higher degree of spatial mismatch with crops compared to *A. mellifera*.

Specifically, *A. cerana* showed a more pronounced decrease in overlap probabilities with several crops, highlighting greater vulnerability to climate-induced changes. Among the 20 crops assessed, *A. cerana* exhibited notable mismatches with at least seven crops, including *C. lanatus*, *F. vesca*, *F. esculentum*, *G. max*, and *G. hirsutum*. These crops showed a consistent reduction in their spatial overlap with *A. cerana*, indicating significant challenges in maintaining pollination services. In contrast, *A. mellifera* demonstrated greater stability in its overlap with most crops, with only moderate mismatches observed for three to four crops, such as *F. vesca* and *G. soja*.

Overall, *A. cerana* is projected to face more serious spatial mismatches across Asia under future climate conditions, as evidenced by its declining overlap probabilities with a wider range of crops. This disparity suggests that *A. mellifera*, despite being non-native, may exhibit greater resilience to changing climatic conditions compared to the native *A. cerana*. The findings highlight the need for targeted conservation and management strategies to mitigate the adverse effects of climate change on *A. cerana* and the crops it pollinates, particularly in regions where these mismatches are most pronounced.

Our findings revealed that *A. mellifera* exhibited a low spatial overlap (D < 0.4) with *A. occidentale*, indicating limited potential interaction between this pollinator and the crop. For *A. cerana*, the crops with a low spatial overlap included *C. sativus*, *G. soja*, and *P. spinosa*. These results highlight potential spatial mismatches between these key pollinators and specific crops, which could have implications for pollination services and agricultural productivity in regions where these crops are cultivated. *A. mellifera* is widely regarded as one of the most critical pollinators for flowering plants [3]. For example, Sagwe et al. [62] reported that *A. mellifera* demonstrated the highest efficiency in pollen deposition among 14 pollinators visiting *P. americana*, ranking in second place for *P. incisa*. In New Zealand, honeybees accounted for over 92% of all avocado visits [63].

Additionally, honeybees and bumblebees are acknowledged as particularly effective pollinators for oilseed rape (*B. napus*), as noted in prior research by Phillips et al. [64]. A global analysis by Garratt et al. [65] revealed that many apple orchards experienced pollination deficits due to insufficient insect pollination. This issue is particularly severe in regions such as Asia, Europe, and South America, as highlighted by Olhnuud et al. [18], who reviewed 48 studies and identified a significant pollination shortfall in apple crops within these areas. However, the mean Schoener’s D overlap in this study for *A. mellifera* across all crops was around 0.63, whereas, for *A. cerana*, it was slightly higher, at approximately 0.67. In contrast, Rahimi et al. [41] conducted a study analyzing the spatial overlap of 394 bee species in Europe and 697 in North America with 41 crops using Schoener’s D statistic. Their findings revealed a mean Schoener’s D of 0.55 for Europe, which was notably higher compared to the value of 0.35 observed for North America.

The crops analyzed in this study are not exclusively pollinated by honeybees, emphasizing the importance of extending similar analyses to other pollinators in Asia. For instance, in Maroua, *E. macrognatha* and *T. fraterna* were identified as the primary pollinators of okra during the 2009 and 2010 cultivation seasons, with their role in pollination confirmed in a subsequent study [66,67]. In Sri Lanka, *T. iridipennis* primarily visited okra flowers for nectar, while *L. atratus* actively collected and transported okra pollen [68]. Similarly, in Indonesia, pollination by three stingless bee species significantly improved okra fruit set, pod size, and seed production [69].

Broader analyses also highlighted the diverse pollinators of crops. For example, Rader et al. [70] found that across 105 pollinator-dependent crops, 93% of the visits were by Hymenoptera, 72% by Diptera, 54% by Lepidoptera, and 51% by Coleoptera. Mango, sunflower, cocoa, and coffee exhibited the greatest diversity of visitor families, ranging between 45 and 59 families. However, most crop pollination is carried out by a small fraction of species; nearly 80% of crop pollination is attributed to just 2% of bee species [71]. For instance, *A. deliciosa* is often visited by insects from the Apidae family, with non-Apis insects accounting for approximately 57% of the visits [72]. For *A. occidentale*, only 13 bee species out of 40 observed have been identified as pollinators [73]. These findings underscore the necessity of conducting spatial overlap analyses for other pollinator groups, as honeybees alone cannot fully account for the diversity of pollination services required by crops in Asia. Climate change may disrupt these critical interactions. This novel framework represents a significant advancement in understanding the impacts of climate change on agricultural and ecological systems.

Also, honeybees may exhibit greater resilience to climate change compared to wild bee species due to their status as managed pollinators [11,74,75]. Beekeeping practices allow humans to actively support and sustain honeybee populations by providing supplemental food, relocating colonies to favorable environments, and implementing disease management strategies. This level of human intervention reduces the impact of climate-driven habitat loss and environmental stressors that wild bees must independently face. Additionally, honeybees have a broad ecological niche [76,77] and a high degree of adaptability, enabling them to persist across a wide range of climatic conditions. Unlike many wild pollinators, which are often more specialized in their foraging habits and dependent on specific plant species, honeybees can utilize a diverse array of floral resources, which makes them less vulnerable to changes in plant distribution caused by shifting climate patterns [77]. Furthermore, because beekeepers can transport colonies across regions to align with crop flowering periods, honeybee populations are not strictly limited by local climate conditions in the same way that wild bees are. These factors collectively suggest that honeybees, as managed pollinators, may have a distinct advantage in adapting to climate change, whereas wild bee populations, lacking human intervention and often exhibiting more specialized ecological requirements, may be at greater risk of decline.

Recent research has increasingly focused on understanding how climate change affects species distribution, including potential mismatches between plants and their pollinators. Since successful interactions require two species to coexist in the same habitat, any divergence in their climatic preferences could lead to disruptions in their spatial overlap under a warming climate [78]. While studies on the future distribution of organisms using SDMs are extensive, research specifically addressing the impact of climate change on plant–pollinator networks remains limited [32].

One of the key challenges is that while it is possible to predict the future distributions of plants and pollinators separately, assessing how these shifts will affect their interactions is more complex. As a result, many researchers rely on simulation models to estimate the potential changes in mutualistic networks [79]. Early studies, such as that by Devoto et al. [80], explored how climate change could lead to species extinction and alter network structures. Schleuning et al. [35] used SDMs to estimate climate change effects on the future distribution of 295 plants, 196 bees, 70 butterflies, and 97 hoverflies. They then analyzed the impact of species decline on network stability by sequentially removing vulnerable species from the interaction matrices. Vizentin-Bugoni et al. [81] introduced an approach that integrates species’ ability to substitute lost connections into coextinction models, assessing network resilience. Their method incorporated rewiring to better estimate pollinator robustness with respect to plant loss, offering a more comprehensive perspective on how pollination networks might adapt to climate change.

A recent study by Rahimi et al. [7] similarly explored the impacts of climate change on plant–pollinator interactions, focusing on geographic co-occurrence to assess potential disruptions. While their innovative methodology provided insights into network-level changes, including the effects of climate change on pollination networks, their approach was primarily binary, analyzing species co-overlapping as a foundation for assessing interaction networks. In contrast, our study adopted a quantitative framework by directly calculating the spatial overlap between pollinators and crops, offering a more nuanced understanding of spatial mismatches under climate change. This distinction allowed us to capture the degree of interaction shifts more comprehensively, advancing the evaluation of climate-induced impacts on pollination services.

In comparison to previous studies, this study successfully addressed spatial mismatches between pollinators and crops by directly integrating spatial data into SDMs. Unlike earlier efforts that relied heavily on simulations or assumptions to estimate changes in plant–pollinator networks under climate change, e.g., [35,81], our study quantified the actual geographic overlap between crops and pollinators. By analyzing spatial discrepancies for each species pair, we overcame the limitations of indirect estimations, providing a more realistic and data-driven evaluation of how climate may affect plant–pollinator interactions.

## 5. Conclusions

This study presented a comprehensive analysis of the spatial interactions between two key honeybee species, *A. mellifera* and *A. cerana*, and 20 pollinator-dependent crops in Asia under both current and future climate scenarios. Using SDMs and Schoener’s D metric, we quantified geographic overlaps and identified significant shifts in pollination dynamics under the SSP585 climate scenario for 2070. Our findings indicate that *A. cerana* is more likely to experience spatial mismatches with crops than *A. mellifera*, suggesting a higher vulnerability to climate-induced changes. Our study also introduced a novel quantitative and spatially explicit framework, improving upon previous methodologies that primarily relied on binary or simulated analyses. While our results highlight the relative resilience of *A. mellifera*, they also emphasize the urgent need for targeted conservation strategies for *A. cerana* and other pollinators. Additionally, extending similar analyses to other pollinator groups will be crucial for capturing the full complexity of pollination services that support crop production in Asia.

Future research should explore how variations in pollinator traits, local adaptation, and landscape heterogeneity influence species interactions under climate change. Investigating potential behavioral and evolutionary responses of honeybee species to shifting environmental conditions could provide deeper insights into their adaptability. Moreover, integrating finer-scale climate and land-use data, as well as experimental validation of predicted shifts, would enhance our understanding of pollinator–crop relationships in a changing climate. This research lays the groundwork for mitigating risks to food security and biodiversity, while opening new avenues for exploring climate-resilient pollination strategies.

## Figures and Tables

**Table 1:** List of studied crops and their pollination dependency. Modest: 10–40% reduction without pollinators; high: 40–90% reduction without pollinators; and essential: >90 reduction % without pollinators.

Pollination Dependency	Scientific Name	Name
Essential	*Citrullus lanatus*	Watermelon
High	*Anacardium occidentale*	Cashew
	*Cucumis sativus*	Cucumber
	*Fagopyrum esculentum*	Buckwheat
	*Mangifera indica*	Mango
	*Prunus avium*	Sour cherry
	*Prunus persica*	Peach
	*Prunus spinosa*	Plum
Modest	*Abelmoschus esculentus*	Okra
	*Castanea sativa*	Chestnut
	*Cocos nucifera*	Coconut
	*Fragaria vesca*	Strawberry
	*Glycine max*	Soybean
	*Glycine soja*	Soybean
	*Gossypium arboreum*	Cotton seed
	*Gossypium hirsutum*	Cotton seed
	*Helianthus annuus*	Sunflower
	*Sesamum indicum*	Sesame
	*Solanum melongena*	Eggplant
	*Vicia faba*	Broad Bean

**Table 2:** Model validation metrics including AUC, BOYCE, and IMAE generated using the MaxEnt algorithm for honeybees and crops.

	AUC	BOYCE	IMAE
*A. mellifera*	0.98	0.97	0.83
*A. cerana*	0.94	0.95	0.82
*A. esculentus*	0.93	0.98	0.84
*A. occidentale*	0.96	0.98	0.89
*C. lanatus*	0.87	0.99	0.73
*C. sativus*	0.91	0.98	0.80
*Castanea sativa*	0.98	0.98	0.90
*F. esculentum*	0.94	0.99	0.82
*M. indica*	0.94	0.99	0.84
*F. vesca*	0.94	0.99	0.78
*C. nucifera*	0.96	0.99	0.88
*G. max*	0.93	0.97	0.84
*G. soja*	0.97	0.89	0.96
*G. arboreum*	0.91	0.87	0.87
*G. hirsutum*	0.92	0.98	0.83
*H. annuus*	0.91	0.99	0.73
*P. persica*	0.95	0.98	0.85
*P. avium*	0.97	0.98	0.87
*P. spinosa*	0.97	0.99	0.88
*S. indicum*	0.91	0.95	0.82
*S. melongena*	0.92	0.99	0.82
*V. faba*	0.94	0.99	0.82

**Table 3:** Number of cells with increased and decreased spatial interactions between mango (*Mangifera indica*) and honeybees across the 22 countries in the study region.

	*A. cerana*		*A. mellifera*	
Country	Decrease	Increase	Decrease	Increase
Bangladesh	47	378	424	1
Bhutan	7	124	37	94
Brunei	0	16	14	2
Cambodia	4	529	79	454
China	3316	30,778	3412	30,682
East Timor	9	23	23	9
India	6472	3438	2712	7198
Indonesia	1561	3327	1891	2997
Japan	190	979	175	994
Laos	136	569	320	385
Malaysia	293	596	400	489
Myanmar	690	1363	1368	685
Nepal	53	435	154	334
North Korea	2	442	0	444
Papua New Guinea	315	912	464	763
Philippines	25	632	148	509
Solomon Islands	2	35	34	3
South Korea	7	318	1	324
Sri Lanka	15	160	28	147
Taiwan	0	99	3	96
Thailand	282	1239	601	920
Vietnam	161	782	587	356

## Data Availability

The data presented in this study are openly available in https://github.com/ehsanrahimi666/Crop-honeybees.git.

## Supplementary Materials

The following supporting information can be downloaded at: https://github.com/ehsanrahimi666/Crop-honeybees.git, accessed on 20 January 2025.
